# A Dibutyl Phthalate Sensor Based on a Nanofiber Polyaniline Coated Quartz Crystal Monitor

**DOI:** 10.3390/s130303765

**Published:** 2013-03-18

**Authors:** You Wang, Pengfei Ding, Ruifen Hu, Jianming Zhang, Xingfa Ma, Zhiyuan Luo, Guang Li

**Affiliations:** 1 National Laboratory of Industrial Control Technology, Institute of Cyber-Systems and Control, Zhejiang University, Hangzhou 310027, China; E-Mails: king_wy@zju.edu.cn (Y.W.); dpfzjuv@126.com (P.D.); jmzhang@iipc.zju.edu.cn (J.Z.); guangli@zju.edu.cn (G.L.); 2 School of Environmental and Material Engineering, Center of Advanced Functional Materials, Yantai University, Yantai 264005, China; E-Mail: xingfamazju@yahoo.com.cn; 3 Computer Learning Research Centre, Royal Holloway, University of London, Egham, Surrey TW20 0EX, UK; E-Mail: zhiyuan@cs.rhul.ac.uk

**Keywords:** QCM, gas sensor, dibutyl phthalate, nanofiber, polyaniline, PACS 07.07.Df (Gas sensor), 77.65.Fs (Quartz resonator), 82.35.Np (Polymers nanoparticles in), 81.07.-b (Nanoscale materials fabrication and characterization)

## Abstract

Dibutyl phthalate (DBP) is a commonly used plasticizer and additive to adhesives, printing inks and nail polishes. Because it has been found to be a powerful reproductive and developmental toxicant, a sensor to monitor DBP in some working spaces and the environment is required. In this work polyaniline nanofibers were deposited on the electrode of a quartz crystal oscillator to form a Quartz Crystal Microbalance gas sensor. The coated quartz crystal and a non-coated quartz crystal were mounted in a sealed chamber, and their frequency difference was monitored. When DBP vapor was injected into the chamber, gas adsorption decreased the frequency of the coated quartz crystal oscillator and thereby caused an increase in the frequency difference between the two crystals. The change of the frequency difference was recorded as the sensor response. The sensor was extremely sensitive to DBP and could be easily recovered by N_2_ purging. A low measurement limit of 20 ppb was achieved. The morphologies of the polyaniline films prepared by different approaches have been studied by SEM and BET. How the nanofiber-structure can improve the sensitivity and stability is discussed, while its selectivity and long-term stability were investigated.

## 1. Introduction

Dibutyl phthalate (DBP), which is soluble in various organic solvents, is a commonly used plasticizer as well as an additive to adhesives, printing inks and nail polishes [1]. At least two decades ago, scientists found that DBP can be a powerful reproductive and developmental toxicant in laboratory animals [1–4], particularly for males [5,6]. The precise mechanism of action is not known, but the pattern of reproductive harm is consistent with other so-called anti-androgens or chemicals that interfere with the male hormones called androgens [1]. Being suspected as an endocrine disrupter, DBP was added to the California Proposition 65 (1986) List of suspected teratogens in November 2006 [7]. Several methods have been reported for the determination of phthalates using fluorescence immunoassay [8], high performance liquid chromatography [9], and mass spectroscopy [10]. Although such techniques provide a low level of detection for phthalates, they are time consuming and have high instrumentation costs.

Quartz crystal microbalance (QCM) sensors have been widely investigated due to their high sensitivity, durability and linearity for mass of the target materials [11–17]. A QCM sensor can be constructed by coating the quartz crystal electrodes surface with a film capable of interaction with the analyte of interest [16,17]. The operating principle of QCM sensors is based on the interaction between the surface of a quartz crystal coated with the sensing materials and the target materials. The Sauerbrey equation was developed for oscillation in air and only applies to rigid mass attached to the crystal [18]. Although a number of polymers have been successfully employed in the coating of QCM sensors [11,15–17], DBP gas sensors based on coated QCM have seldom been investigated. We study herein a QCM sensor with a nanofiber polyaniline film for highly sensitive DBP detection in air. Besides evaluating of the sensor performance, the way in which the nanofiber-structure of polyaniline can be used to improve the response feature of the QCM sensors is also investigated and discussed.

## 2. Experimental Section

### 2.1. Materials

Aniline (AR), dibutyl phthalate (AR), ammonium peroxydisulfate (AR), poly(sodium-*p*-styrene sulfonate) and hydrochloric acid (AR) were commercially available. Aniline was freshly distilled under vacuum prior to use. Deionized filtered water was used in all studies. Ethanol (AR), acetaldehyde (AR), acetone (AR), dimethyl phthalate (AR) and diethyl phthalate (AR) were also all commercially available. AT-cut 6.0 MHz (HC-49/U) quartz crystals with electrodes on both sides were purchased from Hosonic International (Hangzhou) Ltd., China. The crystals were rinsed in ethanol and then deionized water prior to use.

### 2.2. Preparation of Sensors with Nanofiber Polyaniline Film

Polyaniline nanofibers used in this paper were synthesized in dilute aniline aqueous solution, using chemical polymerization methods. According to the method published by Epstein [19], a dilute aniline solution was prepared by adding an equal amount of aniline (*ca.* 8 mM) and ammonium peroxydisulfate to the hydrochloric acid solution, which was then dispersed by ultrasonication. Polyaniline nanofiber was obtained after standing for 24 h. Then a certain volume of the homogenous polyaniline solution was coated on the QCM. After drying for 96 h at room temperature, the nanofiber polyaniline was deposited on the surface of QCM, whose sensing area is 0.5 cm^2^, this forming a QCM sensor modified with a nanofiber-structured polyaniline film.

### 2.3. Preparation of Sensors with Non-Nanofiber Polyaniline Film

In order to contrast with the polyaniline nanofibers sensing film and to study the effect on gas sensing properties of the nanofiber-structured polyaniline, the corresponding polyaniline without a nanofibrous structure was chosen as the contrast sensing material. The polyaniline was prepared by adding aniline (*ca.* 400 mM), ammonium peroxydisulfate (*ca.* 400 mM) and poly(sodium-*p*-styrene sulfonate) (*ca.* 6%, w/w) to a hydrochloric acid solution. The solution of non-nanofibrous polyaniline was obtained after standing for 24 h. A drop-coating method was used to form a polyaniline film on the surface of the electrode.

### 2.4. Charaterization

Scanning electron microscopy (SEM) observation was performed using a Field-Emission Scanning Electron Microscope equipped with an Energy Dispersive Spectrometer (FESEM-EDS, HITACHI S4800, Tokyo, Japan). Brunauer-Emmett-Teller (BET) nitrogen adsorption-desorption data were obtained on a Micromeritics Analyzer (ASAP 2020 V3.01 E analyzer, Micromeritics Instrument Corporation, Norcross, GA, USA) at 77 K. A measuring microscope (107JA precise measuring microscope purchased from Shanghai CSOIF, Co., LTD., Shanghai, China) was used to measure the polyaniline film thickness.

### 2.5. Gas Sensing Experiments

A sensing film coated crystal was used as a sensing QCM while an uncoated crystal was used as a reference QCM. Both of the reference and sensing QCM set in a 500 mL sealed chamber were exposed to DBP or analytical gases for 5 min at 20 degree Celsius. For each vapor sample, 10 mL pure DBP or other gases were left on the bottom of a 1,000 mL glass container for 3 h, so that saturated volatile organic compounds could be extracted in the headspace of the container as the analyte. Appropriate analyte concentrations were obtained in the test chamber by injecting known volumes of headspace gas via a gas tight syringe [16]. Moreover, a small plastic air bag was used to keep the pressure balance. A driving circuit made the QCMs oscillating and output the frequency difference. When an gas to be analyzed was injected into the testing chamber, the frequency difference change was defined as the response of the sensor. The frequency difference between reference and sensing QCMs was measured continuously at 1 second intervals. After the QCM sensor response stabilized, high-purity N_2_ was used to purge the chamber to recover the sensor.

## 3. Results and Discussion

### 3.1. SEM and BET Studies of Sensing Films

**Figure 1.**
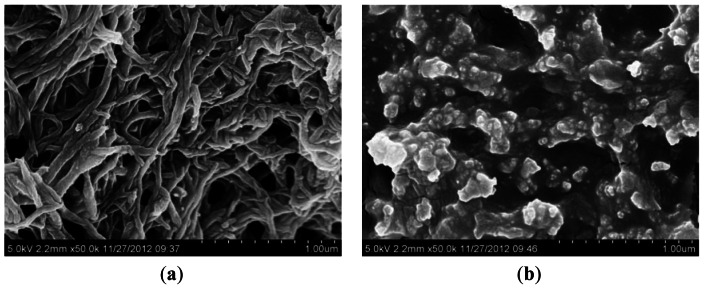
SEM images of the nanofiber-structured (**a**) and non-nanofiber (**b**) polyaniline film on a quartz crystal electrode.

The polyaniline film on the QCM sensor was deposited by platinum on the surface for SEM observation. It can be seen in Figure 1(a) that the nanofiber-structured polyaniline film consists of relatively uniform nanowires with diameters of approximately 60 nm. In comparison, Figure 1(b) shows the morphology of the polyaniline film, where there are no nanofibers, but only agglomerates and particulates.

Nitrogen adsorption and desorption analyses were conducted to investigate the surface area and pore structures of polyaniline films. Table 1 lists the BET data of nanofiber-structured and non-nanofiber polyaniline, indicating the BET surface area of nanofibrous polyaniline was around 175 times larger than that of the contrast non-nanofiber polyaniline film.

### 3.2. Optimization of the Thickness of Nanofiber-Structured Sensing Films

**Figure 2.**
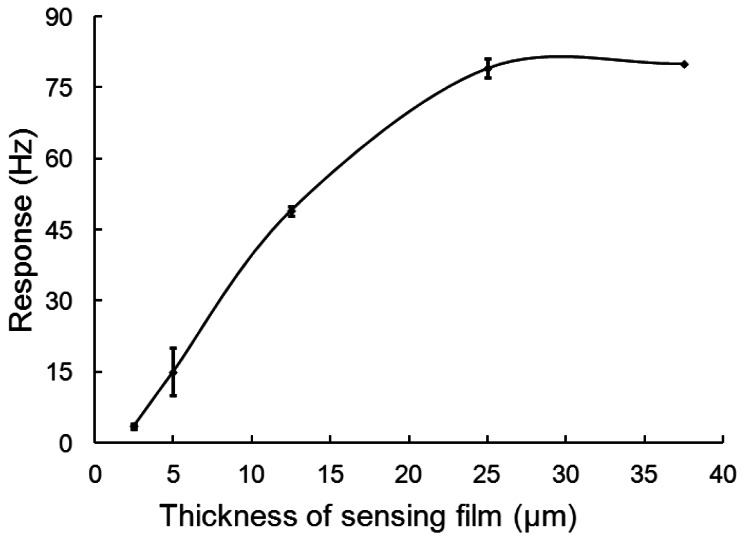
Responses of QCM sensors to 0.2 ppm DBP with different thickness of sensing film.

The thickness of the sensing film on the electrode was a key factor affecting the sensitivity of the sensor, which is controlled by the volume of the homogenous polyaniline solution coated on the QCM. Figure 2 shows the responses of the sensors with different thickness of the sensing films to 0.2 ppm DBP. As the thickness of polyaniline films increased from 2.5 to 25.0 μm, the responses increased dramatically. When the thickness of polyaniline films was more than 25.0 μm, the responses did not increase further. In order to maintain the fastest response, the optimal thickness of polyaniline films was chosen as 25.0 μm.

### 3.3. Sensitivity and Repeatability

The Sauerbrey equation [18] gives the change in the oscillation frequency of piezoelectric quartz (Δ*f*) as a function of the mass (Δ*m*) added to the crystal:

(1) $$\Delta f=-\frac{2f_{0}^{2}}{A\rho_{q}\upsilon_{q}}\Delta m$$

where Δ*f* is the observed frequency change (Hz), *f*_0_ is the fundamental resonant frequency of the crystal, *A* is the active area of the crystal (between electrodes), *ρ_q_* is the density of quartz and *υ_q_* is the shear wave velocity in the quartz.

**Figure 3.**
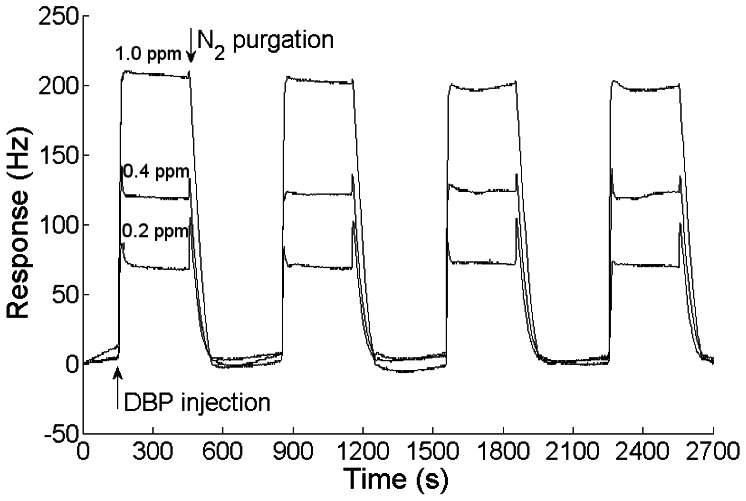
Response cycles of a nanofiber-structured polyaniline film based QCM sensor to different concentrations of DBP purged by high-purity N_2_ at room temperature.

When an analyte is injected into the testing chamber, the sensing film adsorbs the analyte and the sensing QCM's frequency decreases. The response, defined as the change of the frequency difference between the sensing QCM and the reference QCM, increases. The recovery of the sensor could be achieved by high-purity N_2_ purging. Figure 3 illustrates the response cycles of a nanofiber-structured polyaniline based QCM sensor to 0.2, 0.4 and 1.0 ppm DBP and purged by high purity N_2_ at room temperature. The sensor showed a good repeatability for certain DBP concentrations and could be easily recovered by high-purity N_2_ purging. The response time (t_90_) of the sensor was less than 30 s.

**Figure 4.**
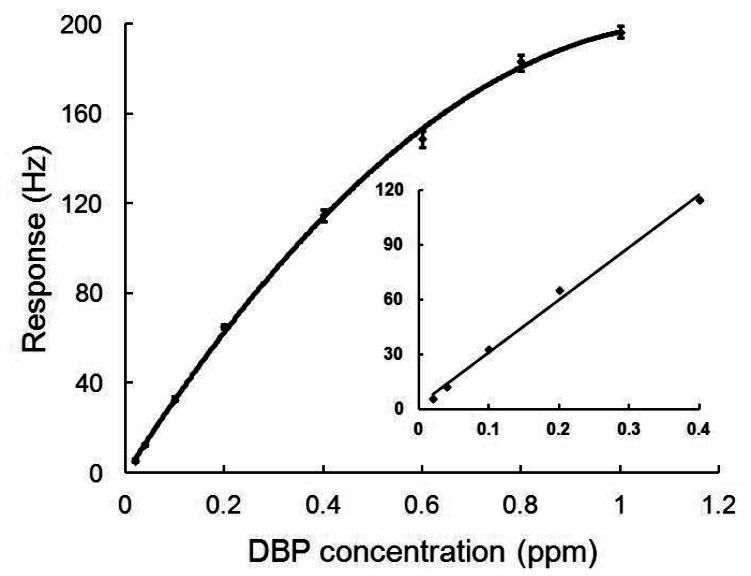
Calibration curve of the nanofiber-structured polyaniline based QCM sensor response to DBP, the inset indicates calibration curve obtained for the linear range.

In Figure 4, the calibration curve of the nanofiber-structured polyaniline based QCM sensor is plotted. The experiments were carried out cyclically with a cleaning step for 5 min in N_2_ and exposure to DBP for 5 min. Each response (R) of the sensor to DBP concentration (C) was repeatedly measured four times. As seen from the Figure 4, responses of the sensor were almost linearly proportional to the DBP concentration in the range from 0.02 to 0.4 ppm. The regression equation is R = 288.16 C + 2.25 with a correlation coefficient of 0.99. The limit of detection (calculated as three times the signal-to-noise ratio) was 0.02 ppm.

### 3.4. Selectivity

**Figure 5.**
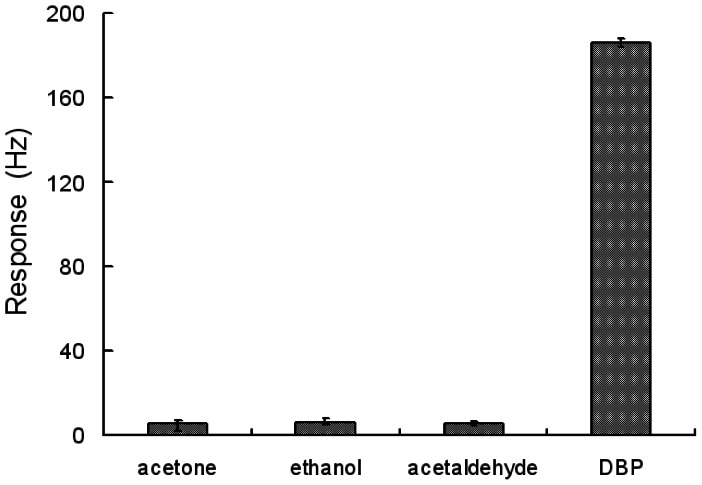
Comparison of the responses of the sensor to different analytes while the concentration of acetone, ethanol and acetaldehyde was 8 ppm and the concentration of DBP was 0.8 ppm.

To investigate the selectivity, the responses of the sensor exposed to acetone, ethanol, acetaldehyde and DBP were measured and analyzed. Although the concentrations of acetone, ethanol and acetaldehyde (8 ppm) were 10 times higher than that of DBP (0.8 ppm), the responses to acetone, ethanol and acetaldehyde were still far less than those to DBP (as shown in Figure 5). The sensor demonstrated an excellent selectivity to DBP from acetone, ethanol and acetaldehyde though they all have carbon-oxygen double bonds or single bonds.

The sensing mechanisms are different according to the characteristics of polyaniline and the target gas [20]. In this work, the interaction of polyaniline with DBP is due to the strong tendency to form hydrogen bonding. As DBP contacted with the polymer, hydrogen bonding was supposed to form between the polyaniline imine groups and the carboxyl groups of DBP. This process made the mass of the film increase, and the frequency of QCM changed. When nitrogen gas was injected into the chamber, hydrogen bonding would be destroyed and DBP was desorbed. Moreover, DBP as a plasticizer has a good compatibility polyaniline polymer. Such characteristics made DBP easily absorbed into polyaniline. Other plasticizers, such as dimethyl phthalate (DMP) and diethyl phthalate (DEP), which have the similar structures to DBP, can also be detected by the QCM sensor. The sensor response ratio to DBP: DEP: DMP was 100: 90: 78.

### 3.5. Long-Term Stability

**Figure 6.**
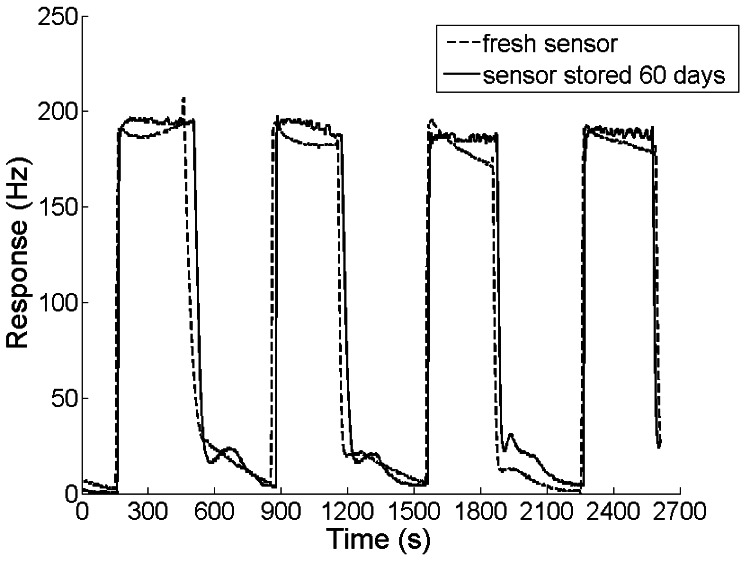
Response cycles to 0.8 ppm DBP. The dashed line represents the response of the immediately fabricated sensor, while the solid line shows the response of the sensor stored in a dry cabinet at room temperature for 60 days.

In order to assess the long-term stability, the response cycles of the sensor used immediately after fabrication were recorded and compared with those of the sensor stored for 60 days. The experimental result is shown in Figure 6. Although the sensor was stored in a dry cabinet at room temperature for 2 months, its response amplitude and recovery remained almost unchanged.

### 3.6. Nano-Structure Effects on the QCM Sensor

In order to investigate the effect of polyaniline nanofiber-structure on the performance of the QCM sensor, polyaniline coated sensors both with and without nanofiber-structure were fabricated and characterized. The morphologies of the polyaniline films with and without nanofiber-structure were illustrated in Figure 1(a,b), respectively.

**Figure 7.**
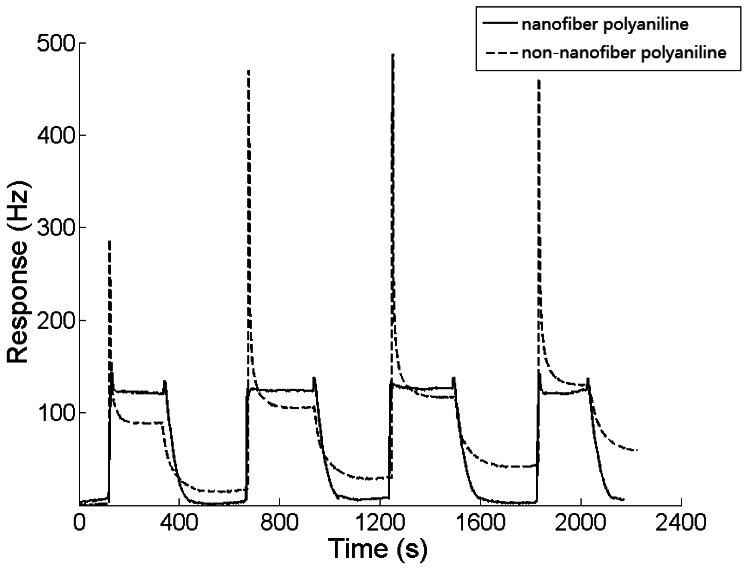
Response cycles to 0.4 ppm DBP of the polyaniline film based sensors with (solid line) and without (dashed line) nanofiber-structure.

The response cycles of both sensors to 0.4 ppm DBP are shown in Figure 7. The average response amplitudes of the two sensors were 117.5 Hz and 87.8 Hz, respectively. The response of the sensor with the nanofiber-structure was 33% larger than the contrast. Not only was the efficient area of the film with nanofiber-structure larger than the contrast, some other nano-scale effects may have also been involved [20,21]. As shown in Figure 7, the baseline of the sensor with a nanofiber-structure was much more stable than the one without nanofiber-structure, while the recovery rate improved significantly. The porosity of the sponge-like nano-structure not only made the analyte adsorption easier, but also made the desorption more thorough.

A large overshoot in the frequency response was observed for the sensor without nanofiber-structure, while there was only a small one for the sensor coated with nanofiber-structured sensing film as shown in Figure 7. In every DBP gas experiment, a high concentration analyte gas was injected into the 500 mL sealed chamber and diffused to equilibrium of a certain concentration in about 5 s, as the diffusion rate is 0.1 L/s, at normal temperature and pressure. As a result of the high concentration of gas being injected close to the QCM sensor, the polyaniline membrane adsorbed much more DBP gas transiently in the beginning, however, as the gas diffused to the bulk medium, desorption happened in the membrane in the meantime, and the amount of DBP adsorbed came into equilibrium, until the adsorption and the desorption were balanced in the end. Because the desorption in non-nanofiber-polyaniline membrane is slower and harder to complete, the frequency of the QCM increased sharply and an overshooting happened due to the fact the quantity of QCM increased sharply when the high concentration of gas was injected. On the contrary, because desorption of nanofiber-polyaniline is faster and easier, the equilibrium of the gas in the membrane can be achieved in a short time, and no overshooting can be detected. The sponge-like nano-structured sensing film reduced the overshoot significantly. This effect proved the proposal by Gardner based on his non-linear diffusion reaction model [22].

## 4. Conclusions

Nanofiber-structured polyaniline films were prepared by a simple non-template method. Based on this film, a QCM sensor to sense dibutyl phthalate was developed and high sensitivity and long term stability were obtained. In comparison with DBP QCM sensor fabricated in a similar way but without nanofiber-structures, the sensitivity and stability of the sensor with nanofiber-structured polyaniline film were significantly improved. Further studies on the QCM sensor response to other plasticizers need to be done and the selectivity of the sensor should be improved. The simple and low-cost method has potential for the development of commercial sensitive DBP sensors for industrial and environmental applications.

## Figures and Tables

**Table 1.** Nitrogen adsorption and desorption analyses of polyaniline films.

**Samples of Polyaniline**	**BET Surface Area** **(m^2^/g)**	**Total Pore Volume** **(cm^3^/g)**	**Average Pore Size** **(nm)**
**Nanofiber-Structured**	14.18	0.21	60.25
**Non-Nanofiber-Structured**	0.08	/	/

## References

[1] Houlihan J., Wiles R. Does a common chemical (dibutyl phthalates) in nail polish and personal care products pose risks to human health? Courtesy of Environmental Working Group. http://www.health-report.co.uk/phthalates.html.

[2] Salazar V., Castillo C., Ariznavarreta C., Campon C., Tresguerres J.A.F. (2004). Effect of oral intake of dibutyl phthalate on reproductive parameters of long evans rats and pre-pubertal development of their offspring. Toxicology.

[3] Ema M., Harazono A., Miyawaki E., Ogawa Y. (1997). Embryolethality following maternal exposure to dibutyl phthalate during early pregnancy in rats. Bull. Environ. Contam. Toxicol..

[4] Ema M., Miyawaki E., Kawashima K. (2000). Effects of dibutyl phthalate on reproductive function in pregnant and pseudopregnant rats. Reprod. Toxicol..

[5] Higuchi T.T., Palmer J.S., Gray L.E., Veeramachaneni D.N.R. (2003). Effects of dibutyl phthalate in male rabbits following in utero, adolescent, or postpubertal exposure. Toxicol. Sci..

[6] Howdeshell K.L., Furr J., Lambright C.R., Rider C.V., Wilson V.S., Gray L.E. (2007). Cumulative effects of dibutyl phthalate and diethylhexyl phthalate on male rat reproductive tract development: Altered fetal steroid hormones and genes. Toxicol. Sci..

[7] Wiki: Dibutyl phthalate. http://wapedia.mobi/en/Dibutyl_phthalate.

[8] Zhang M., Wang Q., Zhuang H. (2006). A novel competitive fluorescence immunoassay for the determination of dibutyl phthalate. Anal. Bioanal. Chem..

[9] Wu G.J.C. (1991). Determination of eleven phthalate esters using HPLC and UV diode-array detector with liquid-liquid extraction or on line preconcentration. J. Environ. Sci. Health Part A.

[10] Berset J.D., Etter-Holzer R. (2001). Determination of phthalates in crude extracts of sewage sludges by high-resolution capillary gas chromatography with mass spectrometric detection. J. AOAC Int..

[11] Matshuguchi M., Kadowaki Y., Tanaka M. (2005). A QCM-based NO_2_ gas detector using morpholinefunctional cross-linked copolymer coatings. Sens. Actuators B Chem..

[12] Koshets I.A., Kazantseva Z.I., Shirshov Y.M., Cherenok S.A., Kalchenko V.I. (2005). Calixarene films as sensitive coating for QCM-based sensors. Sens. Actuators B Chem..

[13] Vilaseca M., Yagüe C., Coronas J., Santamaria J. (2006). Development of QCM sensors modified by AlPO4–18 films. Sens. Actuators B Chem..

[14] Sasaki I., Tshuchiya H., Nishioka M., Sadakata M., Okubo T. (2002). Gas sensing with zeolite-coated quartz crystal microbalances—Principal component analysis approach. Sens. Actuators B Chem..

[15] Mirmohseni A., Oladegaragoze A. (2003). Construction of a sensor for determination of ammonia and aliphatic amines using polyvinylpyrrolidone coated quartz crystal microbalance. Sens. Actuators B Chem..

[16] Li G., Zheng J., Ma X., Sun Y., Fu J., Wu G. (2007). Development of QCM trimethylamine sensor based on water soluble polyaniline. Sensors.

[17] Zheng J., Li G., Ma X., Wang Y., Wu G., Cheng Y. (2008). Polyaniline-TiO_2_ nano-composite-based trimethylamine QCM sensor and its thermal behavior studies. Sens. Actuators B Chem..

[18] Sauerbrey G. (1959). The use of quartz oscillators for weighing layers and for micro-weighing. Z. Phys..

[19] Chiou N.-R., Epstein A.J. (2005). Polyaniline nanofibers prepared by dilute polymerization. Adv. Mater..

[20] Ji S., Li Y., Yang M. (2008). Gas sensing properties of a composite composed of electrospun poly(methyl methacrylate) nanofibers and *in situ* polymerized polyaniline. Sens. Actuators B Chem..

[21] Tischner A., Maier T., Stepper C., Köck A. (2008). Ultrathin SnO_2_ gas sensors fabricated by spray pyrolysis for the detection of humidity and carbon monoxide. Sens. Actuators B Chem..

[22] Gardner J.W. (1990). A non-linear diffusion-reaction model of electrical conduction in semiconductor gas sensors. Sens. Actuators B Chem..

